# Epigenetic Mechanisms Involved in Hcv Infection and Human HCC

**DOI:** 10.3390/ijms262412045

**Published:** 2025-12-15

**Authors:** Ximenya Glauce da Cunha Freire Lopes, Roque Ribeiro da Silva Júnior, Fernando Liberalino Fernandes, Laura Andrade Custódio de Oliveira, Vania Sousa Andrade, Fabiana Lima Bezerra, Josélio Maria Galvão de Araújo, Jenner Chrystian Veríssimo de Azevedo, Thales Allyrio Araújo de Medeiros Fernandes, José Veríssimo Fernandes

**Affiliations:** 1 Postgraduate Program in Parasitic Biology (PPGBP), Federal University of Rio Grande do Norte (UFRN), Natal 59078-900, Brazil; ximenya.glauce@gmail.com (X.G.d.C.F.L.); vsandrade@cb.ufrn.br (V.S.A.); fabiana.lima@ufrn.br (F.L.B.); joselio.araujo@ufrn.br (J.M.G.d.A.); verissimo.fernandes@ufrn.br (J.V.F.); 2 Multicenter Graduate Program in Physiological Sciences (PPGMCF), Faculty of Health Sciences (FACS/UERN), State University of Rio Grande do Norte (UERN), Mossoro 59610-090, Brazil; thalesallyrio@uern.br; 3 Multicenter Graduate Program in Biochemistry and Molecular Biology (PMBqBM), Faculty of Health Sciences (FACS/UERN), State University of Rio Grande do Norte (UERN), Mossoro 59610-090, Brazil; fernandoliberalino@alu.uern.br; 4 Department of Medicine, Federal University of Rio Grande do Norte (UFRN), Natal 59078-900, Brazil; laura.andrade.093@ufrn.edu.br (L.A.C.d.O.); jennerazevedo@hotmail.com (J.C.V.d.A.)

**Keywords:** Hepacivirus, Epigenomics, RNA

## Abstract

Hepatitis C virus (HCV) infection remains a major global health challenge and often progresses to chronic liver disease and hepatocellular carcinoma (HCC). Growing evidence indicates that epigenetic regulation mediated by non-coding RNAs plays a critical role in viral pathogenesis and tumor development. This review provides an integrated overview of the functions of microRNAs (miRNAs), long non-coding RNAs (lncRNAs), and circular RNAs (circRNAs) in HCV-induced liver injury. We highlight the dual roles of these molecules, demonstrating how some ncRNAs promote viral replication, whereas others act as tumor suppressors that become dysregulated during infection. Particular emphasis is placed on interaction networks in which lncRNAs and circRNAs function as molecular sponges for miRNAs, thereby modulating signaling pathways essential for hepatic homeostasis. Disruption of these networks contributes to a pro-inflammatory and pro-tumorigenic microenvironment. Finally, we discuss the potential of these transcripts as diagnostic biomarkers and as emerging therapeutic targets in HCV-associated HCC.

## 1. Introduction

The hepatitis C virus (HCV) was discovered in 1989 [1], and following its molecular characterization, it was classified within the Flaviviridae family, genus Hepacivirus [2,3]. It is an enveloped virus with a single-stranded positive-sense RNA genome enclosed within an icosahedral capsid [4,5]. The HCV remains a major global public health concern, with an estimated 71 million individuals chronically infected worldwide. Infection frequently progresses to chronic hepatitis and, over time, may lead to liver cirrhosis and hepatocellular carcinoma (HCC) [6].

The HCV genome is approximately 9.6 kb in length and contains a single open reading frame that encodes a precursor polyprotein. This polyprotein is processed by viral and host proteases to generate three structural proteins (core protein C and the envelope glycoproteins E1 and E2) and seven nonstructural proteins (p7, NS2, NS3, NS4A, NS4B, NS5A and NS5B) [7]. The high error rate of the viral RNA-dependent RNA polymerase, combined with selective pressures imposed by the host immune system, promotes viral evolution through mechanisms of genetic variation that produce distinct genotypes. As a result of the elevated mutation rate, numerous genetically related viral variants known as quasispecies are continuously generated during infection [8].

The viral nucleocapsid is approximately 45 nm in diameter and is surrounded by a lipid envelope, forming a particle that measures between 56 and 65 nm in total diameter [9]. The envelope contains glycoprotein spikes composed of E1 and E2 heterodimers, which mediate viral entry into host cells [10,11,12]. The E2 glycoprotein binds to the host cell surface through interactions with scavenger receptor class B type I (SR-BI) and CD81 [13]. Most viral particles circulate as lipoviral particles. These are virions associated with low-density lipoproteins (LDL), very low-density lipoproteins (VLDL), and apolipoproteins A1, B, C, and E. These lipid components, together with the E1 and E2 glycoproteins in the viral envelope, protect the virus from neutralizing antibodies. They also contribute to immune evasion [14,15,16].

The evolutionary process of HCV has resulted in extensive genetic diversity, with eight genotypes and more than ninety subtypes currently identified, some of which are geographically restricted. This high genetic variability may reduce the effectiveness of antiviral therapies and complicates the development of safe and effective vaccines aimed at global viral elimination [17].

In general, the acute phase of HCV infection is clinically silent and does not present with signs or symptoms. Only about twenty percent of patients develop manifestations such as fatigue, anorexia, nausea, vomiting, abdominal pain, and jaundice. The clinical course of the acute phase is usually benign and rarely requires hospitalization. Despite this favorable presentation, most individuals infected with HCV progress to chronic infection characterized by persistent viral replication [18].

Chronic hepatitis C is defined by the persistence of viral RNA in the bloodstream for more than six months after the onset of acute infection. This condition develops in approximately fifty-five to eighty-five percent of patients who, after an often-asymptomatic acute phase, progress to chronic disease. Spontaneous viral clearance is uncommon, and progression to fibrosis, steatosis, cirrhosis, end-stage liver disease, and hepatocellular carcinoma (HCC) may occur [19,20,21]. A large cohort study demonstrated that host factors such as sex, age, and HLA profile, as well as viral factors including genotype and HIV co-infection, influence infection outcomes. Younger age, female sex, and the presence of symptoms during the acute phase were associated with a higher probability of spontaneous viral clearance [18,22].

Chronic HCV infection is characterized by persistent inflammation that contributes to progressive liver damage with disturbances in lipid metabolism, steatosis, cirrhosis, and fibrosis. Alterations in immune responses, together with genetic and epigenetic modifications, create an environment conducive to the development of severe hepatic disease. These conditions promote the proliferation of infected cells, inhibit apoptosis, and stimulate neoangiogenesis, which collectively support the initiation and progression of HCC [23].

Epigenetic mechanisms involving DNA methylation, histone modifications, and non-coding RNA (ncRNA) activity are fundamental regulators of gene expression and can activate or suppress the transcription or translation of both cellular and viral genes. Aberrant activity or dysregulated expression of epigenetic components plays a critical role in the pathogenesis of numerous diseases, including inflammatory and malignant conditions [24]. One study demonstrated that eleven tumor suppressor genes are hypermethylated and downregulated in HCV-associated HCC-derived cells, with protein expression suppressed at the transcriptional level [25].

Chronic HCV infection promotes persistent inflammation, generating a microenvironment that favors the activation of epigenetic mechanisms, including the induction or repression of ncRNA expression. These molecules function as central regulators of intracellular biochemical pathways that may drive malignant transformation and contribute to the initiation and progression of HCV-induced hepatocellular carcinoma [26,27,28]. In this review, we summarize the most recent scientific advances regarding the role of ncRNAs as regulators of intracellular biochemical processes during chronic HCV infection. We describe how these molecules exacerbate virus-induced liver injury, potentially promoting the onset and progression of HCC.

## 2. Methodology

This comprehensive review was conducted in accordance with the methodological procedures described in the studies by Figueirôa et al. [29] and Ribeiro et al. [30], using the PubMed, Embase and Web of Science databases. For this purpose, descriptors were selected from the Medical Subject Headings (MeSH) and Emtree platforms. The following descriptors were used: Hepacivirus, Epigenomics and RNA.

Eligibility criteria included original articles published to date that examined the relationship between chronic HCV infection and epigenetic mechanisms, including miRNAs, lncRNAs, circRNAs, DNA methylation and histone modifications. Exclusion criteria comprised undergraduate and graduate theses, editorials, preprints, non-peer-reviewed materials, articles not available in full text and studies addressing other viral hepatitis infections or ncRNAs unrelated to HCV or HCC.

**Figure 1:**
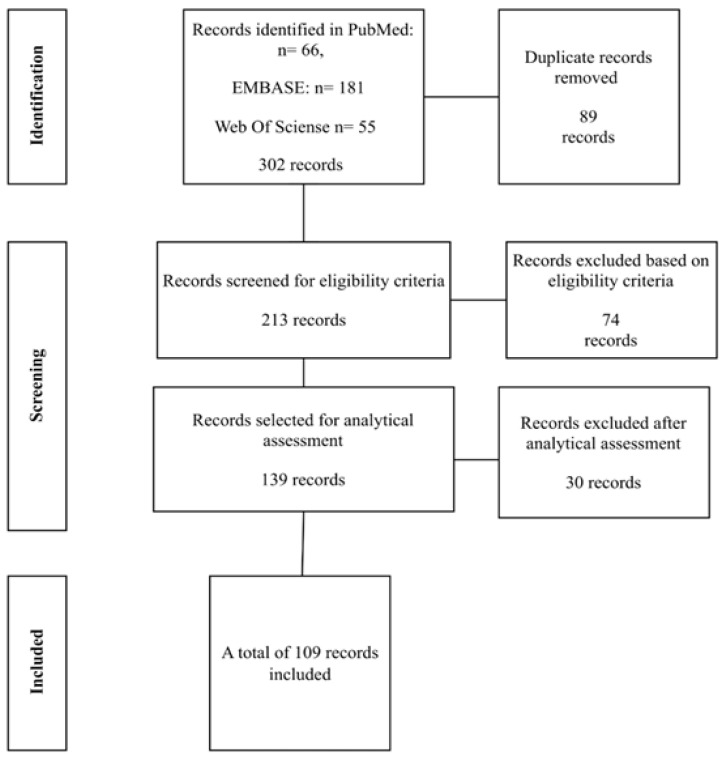
Flowchart of studies selected for the review. Source: author (2025).

A total of 302 articles were identified across the databases. After the removal of 89 duplicate records, 213 studies remained and were screened according to the eligibility criteria. At this stage, 74 articles were excluded. Of the 139 remaining studies, an analytical assessment was performed, resulting in the exclusion of an additional 30 articles. Consequently, 109 studies were included in the review, as shown in Figure 1 below.

## 3. Results

### 3.1. Role of Non-Coding RNAs

Noncoding RNAs (ncRNAs) are a heterogeneous class of transcripts that are not translated into proteins. They participate directly or indirectly in multiple intracellular biological processes and regulate functions that are essential for cellular homeostasis. Dysregulation of these transcripts can activate pathological mechanisms in humans, particularly those associated with cancer [31]. NcRNAs operate within gene regulatory networks, interacting with one another as well as with DNA, mRNA, and proteins [32]. As a result, ncRNAs play increasingly significant roles in both physiological and pathophysiological processes in human cells [33]. Sequence analysis of enhancer elements can also provide valuable insights, since these regions exert a critical influence on gene expression and its dysregulation in disease [34].

Although no universally accepted definition of ncRNAs exists, they are commonly classified by transcript length into three major groups: microRNAs (miRNAs), long noncoding RNAs (lncRNAs), and circular RNAs (circRNAs) [35,36]. MiRNAs typically range from nineteen to twenty-five nucleotides in length, whereas lncRNAs exceed two hundred nucleotides. All of these molecules regulate gene expression at transcriptional, post-transcriptional, translational, and post-translational levels by selectively binding to proteins and to target DNA and mRNA sequences [33,34,35]. CircRNAs share certain structural and functional similarities with lncRNAs; however, their 5′ and 3′ termini are covalently joined to form a closed loop. They function as sponges for RNA and proteins and modulate gene expression and alternative splicing [35,36,37,38,39,40].

MiRNAs regulate gene expression at the mRNA and protein levels through post-transcriptional mechanisms by inducing mRNA degradation or inhibiting its translation into protein [41,42]. Accordingly, mRNAs can be silenced through three main mechanisms: degradation, destabilization caused by shortening of the poly A tail, and reduced translational efficiency by ribosomes [43,44]. Nucleolar miRNAs also exist and function as guides for the chemical modification of ribosomal RNA (rRNA), a process essential for ribosomal structure, ribosomal function, and efficient protein synthesis [45].

Overexpression of several microRNAs has been reported in HCV-induced HCC cells, where they contribute to oncogenic processes including cell cycle progression, increased cellular proliferation, and loss of cell adhesion. Other microRNAs suppress antitumor immune responses. Thus, specific microRNAs act as dual mediators that support both HCV replication and the progression of liver disease, including hepatocellular carcinoma [46].

LncRNAs have gained particular prominence among ncRNAs because they exhibit cell-type specific expression and regulate numerous cellular events, including proliferation, differentiation, and regeneration, in addition to a wide range of physiological processes [38,41,47]. CircRNAs, due to their closed-loop structure, display high molecular stability and exert critical regulatory effects on gene expression [48,49]. They can interact with RNA-binding proteins that play essential roles in tumorigenesis and metastasis, thereby influencing gene expression and altering processes associated with tumor initiation and progression [50]. CircRNAs also function as sponges for miRNAs, neutralizing cis regulatory gene effects, mediating protein binding, and, in some cases, contributing to protein coding. These features confer substantial regulatory potential to circRNAs in eukaryotic cells and enable their involvement in human disease development [51,52].

Since their discovery, ncRNAs have emerged as essential regulators of multiple biological processes in the human body, including immune system function. Their dysregulation has been implicated in the development of numerous diseases, particularly cancer [53,54]. LncRNAs and circRNAs are now increasingly recognized as significant contributors to the pathophysiology of many human disorders, especially malignant disease [31].

### 3.2. Participation of miRNAs in HCV Infection

Like all viruses, HCV depends on host cells for replication and therefore recruits host cellular factors to its advantage. These factors include miRNAs that can be manipulated by the virus to create an environment favorable to its replication. Numerous miRNAs are dysregulated in HCV-infected cells, and this dysregulation may modulate viral replication directly or indirectly, with important implications for the pathogenesis of hepatitis C, including hepatocellular carcinoma [55]. In contrast, infected cells may also induce the expression of miRNAs that suppress HCV replication, thereby reducing the likelihood that hepatic lesions will progress to severe liver disease, including cancer.

A well-characterized example of a miRNA that promotes HCV replication is the liver-specific miR 122, which is required by the virus for efficient replication and is therefore highly expressed during infection. The interaction between miR 122 and HCV deviates from the canonical function of miRNAs, which typically bind to the 3 prime untranslated region (UTR) of mRNAs. In this context, miR 122 binds to two sites within the 5 prime UTR of the viral genome and enhances replication. MiR 122 is essential for efficient HCV replication because it stabilizes the viral RNA and increases its translation through induced structural alterations. This interaction directly influences the liver tropism of HCV and contributes to viral pathogenesis [56].

Conversely, several miRNAs inhibit HCV replication by acting at different stages of the viral life cycle and thereby shape disease pathogenesis. Table 1 summarizes the functions of selected miRNAs involved in HCV infection that are discussed in this review.

MicroRNA signatures have been proposed as prognostic tools for assessing the risk of HCC progression in patients with chronic HCV infection. Individually, miR 618 and miR 650 demonstrated sensitivities of sixty-four percent and seventy-two percent and specificities of sixty-eight percent and fifty-eight percent, respectively. When used in combination, miR 618 and miR 650 achieved a sensitivity of fifty-eight percent and a specificity of seventy-five percent, resulting in significantly higher predictive performance compared with the traditional diagnostic approach based on alpha-fetoprotein (AFP) levels [46].

MiR 10a regulates several genes involved in hepatic metabolism and is upregulated by HCV, with markedly elevated expression in advanced chronic hepatitis C and hepatocellular carcinoma. Increased miR-10a expression leads to downregulation of the circadian rhythm gene Bmal1, which in turn suppresses lipid synthesis genes such as sterol regulatory element binding protein (SREBP1), fatty acid synthase (FASN), and SREBP2, as well as gluconeogenic pathways required for viral replication. Reduced Bmal1 expression is associated with elevated serum alanine aminotransferase levels and with progression to liver fibrosis and HCC. This disruption of metabolic adaptation further exacerbates hepatic metabolic abnormalities in patients with hepatocellular carcinoma [57].

The CD81 receptor is expressed on immune cells and plays essential roles, particularly in B cells [58], whereas tight junction proteins such as claudin 1 (CLDN1) and occludin (OCLN) are key components required for HCV entry into hepatocytes [59]. Specific miRNAs, including miR 194, miR 548, miR 182, and miR 200c, negatively regulate the expression of these genes, thereby preventing HCV entry and reducing viral replication [55]. MiR 194 and miR 548 act cooperatively to inhibit CD81 expression and consequently reduce viral infectivity [60,61]. MiR 182 and miR 200c suppress the expression of CLDN1 and OCLN, respectively, which also contributes to decreased viral replication [62,63].

Other miRNAs, such as miR 196, miR 296, miR 351, miR 431, and miR 448, interact directly with the viral genome and inhibit replication. In addition, miR-199a and miR let-7b bind to the 5 prime untranslated region (UTR) of the viral genome and silence the gene encoding the nonstructural protein NS5B, whereas miR-181c suppresses viral genes encoding glycoprotein E1 and NS5A. Collectively, these miRNAs interfere with multiple steps of HCV replication [64,65]. These findings indicate that such miRNAs, either individually or in combination, warrant investigation as potential therapeutic agents, although several technical challenges must first be addressed [66].

A study reported that miR-215-5p, miR-10b-5p, and let-7a-5p are upregulated during chronic HCV infection and together suppress the expression of the oncogene reticulocalbin 1 (RCN1) [61]. RCN1 plays a crucial role in the development of hepatocellular carcinoma by regulating the transport of insulin-like growth factor (IGF), its uptake by IGF binding proteins (IGFBPs), and protein metabolism [67].

Analysis of the molecular interactions among four key regulators of lipid homeostasis, miR 24, miR 122, miR 223, and PCSK9, in HCV-infected patients who achieved cure following interferon-based therapy or first-generation direct-acting antivirals demonstrated that levels of miR 24, miR 223, and PCSK9 increased significantly. Meanwhile, levels of miR 122, an inducer of HCV replication, decreased [68]. These findings suggest that miR-24, miR-223, and PCSK9 may contribute to controlling viral infection.

Serum levels of miR-122 and miR-486 are elevated in chronically HCV-infected patients compared with healthy individuals. In physically active patients, the expression of these miRNAs correlates positively with increased alanine aminotransferase (ALT), aspartate aminotransferase (AST), fibrosis, and inflammatory responses, although no association with higher viral load has been observed. The precise role of miR 486 in HCV infection remains unclear, but it may modulate cellular processes that influence viral replication and disease progression [69].

Expression levels of miR 122 and miR 486 correlate strongly with physical activity, muscle pain and fatigue in HCV infected patients. Higher expression of these transcripts is also associated with changes in body mass index and with the stage of liver fibrosis. These findings reinforce the clinical recommendation that patients with HCV infection limit strenuous physical activity [69].

Although it remains unclear whether miRNA dysregulation is a cause or a consequence of hepatocellular carcinoma, distinct miRNA expression patterns have been documented across different stages of liver injury, including HCC. Elevated serum levels of miR 122, miR 21 and miR 223 have been reported in patients with HCC, and these miRNAs appear to contribute to malignant transformation and tumor initiation [70].

Expression levels of miR 21, miR 122 and miR 222 have been examined in three groups of chronically HCV infected patients: those without cirrhosis, those with cirrhosis and those with HCC. Serum levels of miR 21 and miR 222 increased progressively with the severity of hepatic lesions and with progression to HCC. MiR 21 downregulates PTEN expression, eliminating its tumor suppressor activity and releasing inhibition of the PI3K Akt pathway. This promotes increased cellular proliferation and survival, thereby facilitating the initiation and progression of HCC [70].

Serum levels of miR-122, miR-335, and miR-483 have been evaluated in ninety patients with HCV-associated hepatocellular carcinoma and ninety chronically HCV-infected patients with non-malignant hepatic lesions. MiR 122 and miR 483 were upregulated in HCV-associated HCC, whereas miR 335 was downregulated. Mechanistically, miR-335 inhibits expression of ROCK1, a gene that promotes cellular proliferation, tumorigenesis, and metastasis. Receiver operating characteristic analysis demonstrated that miR 483 displayed the highest diagnostic accuracy, achieving one hundred percent detection capability [71]. These findings suggest that miR 335 exerts a protective effect against HCV-induced HCC, whereas miR 483 may serve as a potential biomarker for disease progression.

Expression levels of miR-139-5p, miR-193a-5p, and miR-940 are reduced in hepatocellular carcinoma, whereas expression of their shared target gene, the oncogene SPOCK1, is increased. SPOCK1 encodes the core protein of a proteoglycan that contributes to dysregulation of the cell cycle, inhibition of apoptosis, impaired DNA repair, and enhanced metastatic potential. In vitro and in vivo assays demonstrated that forced expression of miR-139-5p, miR-193a-5p, and miR-940 suppresses viability and invasion of HCC cells, promotes apoptosis, and inhibits tumor growth. Conversely, SPOCK1 overexpression increases proliferation and invasion and reduces apoptosis [72]. These findings demonstrate that these three miRNAs collectively function as critical suppressors of HCC initiation, progression, and invasion.

Expression of miR-198 is significantly reduced in chronic HCV infection and in hepatocellular carcinoma. This reduction correlates with advanced clinical stage, tumor capsular infiltration, and metastasis. Targets of miR-198 include genes that regulate cell proliferation and migration [73]. Forced expression of miR-198 in hepatoma cells inhibits the expression of the signal transducer genes c-MET and CDK4, which promote proliferative pathways, and increases the expression of E-cadherin and claudin-1, which maintain cellular adhesion [73,74]. Thus, under physiological conditions, miR 198 functions as a suppressor of hepatocellular carcinoma by inhibiting genes involved in proliferation and migration while activating genes necessary for cell adhesion.

Upregulation of the demethylating enzyme TET1 inhibits HCV-induced tumorigenesis, proliferation, migration, and invasion; reduces inflammatory mediators in hepatocellular carcinoma; and promotes autophagy and apoptosis. TET1 activates miR 34a expression by demethylating its promoter, and miR 34a subsequently inhibits expression of the BACH1 gene, whose product disrupts homeostasis, increases oxidative stress, and contributes to malignant processes. Inhibition of miR-34a results in increased inflammatory mediators and reduced autophagy and apoptosis in the presence of TET1, whereas silencing of BACH1 reverses these effects even when miR-34a is inhibited [75]. These findings indicate that miR-34a prevents the progression of hepatocellular carcinoma by suppressing BACH1 expression.

Serum levels of miR 223 are approximately twofold lower in patients with hepatocellular carcinoma than in HCV-infected patients without tumors, used as controls. An inverse pattern is observed for miR 19a, whose serum levels are elevated in HCC and correlate with increased tumor size [76]. These findings indicate that miR 223 exerts a suppressive role, whereas miR 19a promotes malignant progression in HCV-induced hepatocellular carcinoma.

MiR 19a has a complex and sometimes contradictory role in HCV infection. Although some studies suggest that it may inhibit viral replication, others indicate that it contributes to liver fibrosis, potentially through activation of STAT3, a transcription factor involved in the proliferation and survival of hepatic stellate cells. MiR-19a silences the SOCS3 gene in stellate cells, allowing activation of the STAT3-mediated TGF-beta signaling pathway and enhancing the expression of fibrogenic genes [77]. These findings support the view that miR-19a functions as an inducer of liver fibrosis in chronically HCV-infected patients.

Serum levels of miR 21, miR 122, and miR 222 have been evaluated in chronically HCV-infected patients with and without malignant disease. MiR-122 levels in patients with non-malignant lesions were higher than in those with HCV-associated hepatocellular carcinoma [78]. This indicates that miR 122, although required for viral replication, is not directly correlated with malignant transformation. Receiver operating characteristic analysis showed that although all three miRNAs were elevated, only miR-21 and miR-222 exhibited sufficient sensitivity and specificity to be considered markers of progression from non-malignant liver lesions to hepatocellular carcinoma. Thus, elevated serum miR-122 levels do not correlate with HCV-associated malignant progression [79].

Elevated serum miR 222 levels in chronic HCV infection correlate with viral pathogenesis, increased severity of hepatic lesions and progression to hepatocellular carcinoma. MiR 222 promotes HCV induced tumor progression by inhibiting the expression of p27 and p57, thereby preventing cell cycle arrest at the G1 S transition. This causes cell cycle dysregulation, loss of DNA repair capacity, accumulation of mutations and activation of pathways that lead to cellular immortalization and malignant transformation. MiR 222 therefore contributes to increased proliferation and reduced apoptosis, facilitating invasion and metastasis of hepatocellular carcinoma cells [78]. These findings indicate that miR 222 has high oncogenic potential and that its elevated expression represents a significant risk factor for HCC progression in chronically HCV infected patients.

MiR-224 has also been implicated in the progression of HCV-induced hepatic injury to hepatocellular carcinoma by targeting the 3prime untranslated region of the glycine N-methyltransferase (GNMT) gene and suppressing its expression. Loss of GNMT tumor suppressor function results in unchecked activation of the mTOR signaling pathway, promoting HCC cell proliferation, motility, and tumor invasion [80].

Although circulating miRNAs represent an important scientific advance and hold considerable promise as diagnostic and prognostic biomarkers for diseases including cancer, quantification based on a single transcript presents methodological limitations and potential technical bias. These limitations include low specificity and poor reproducibility. Furthermore, pre analytical variables such as the type of biological fluid used (serum or plasma), the choice of anticoagulant and centrifugation conditions can substantially affect the detected miRNA profile [81].

**Table 1:** List of miRNAs cited in this review, distributed according to the function they perform in HCV-infected liver cells leading to progression to HCC.

**MiRNA**	**Protective Function Against the Development of HCC**	**References**
miR-194 e miR-548	Inhibit viral replication by reducing the expression of the CD81 receptor.	[60,61]
miR-182 e miR-200c	Inhibits the expression of CLDN1 and OCLN, respectively.	[62,63]
miR-199a miR-let-7b miR-181c	Binds to the 5′ UTR, inhibiting the translation of viral mRNA. Silences the non-structural protein NS5B gene. Silences the E1 glycoprotein and NS5B genes	[64,65]
hsa-miR-215-5p hsa-miR10b-5p hsa-let-7a-5p	It acts together by inhibiting the expression of the RCN1 oncogene whose product suppresses the apoptosis of HCC cancer cells.	[82]
miR-335	Inhibits the expression of ROCK1 whose product induces cell proliferation, tumorigenesis and metastasis.	[71]
mir-139-5p miR-139a-5p miR-940	Inhibit SPOCK1 expression reducing proliferation and invasion of tumor cells.	[72]
miR-198	Inhibits c-MET which leads to inhibition of the p44/42 MAPK pathway, increasing the expression of E-cadherin and claudin-1 and decreasing cell proliferation and migration.	[73,74]
miR-34a	Inhibit BACH1 expression increasing autophagy and apoptosis of cancer cells.	[75]
miR-9-5 p	It acts by suppressing the expression of the BGH3 oncogene.	[83]
miR-124	Tumor suppressor action by inhibiting AQP3 expression.	[84]
miR516a-5p	Acts by inhibit TRAF6 e MAPK11 and the NF-κB and MAPK pathways reducing inflammation induced apoptosis of tumor cells.	[85]
miR-145-5P	Tumor suppressor action by inhibit the expression of SPATS2 and increased p21 and p27 which inhibits cell proliferation and migration.	[86,87]
**MiRNA**	**Inducing function of HCC initiation and progression**	**References**
miR-122	It binds to two sites in the 5′ UTR of the viral genome to promote viral replication.	[55]
miR-10a	Downregulates the liver circadian rhythm gene Bmal1 causing exacerbation of abnormal hepatic metabolism.	[57]
miR-21	Inhibits PTEN which leads to activation of PI3K/AKT/mTOR which promoting cell survival and proliferation	[70]
miR-19a	It acts by silencing the SOCS3 gene in HSCs to allow activation of the STAT3-mediated TGF-β signaling pathway.	[77]
miR-222	Inhibits p27 and prevents its function of negative regulator of the AKT, promoting cell survival and proliferation.	[78]
miR-224	Inhibits GNMT, abolishing its tumor suppressor function by ceasing to inhibit the mTOR pathway, leading to cell proliferation.	[80]
miR-152	Inhibits the Wnt1 cellular signaling pathway preventing HCC initiation.	[88]
miR-184	downregulates the tumor suppressor SOX7 and upregulates the oncogene c-MYC, promoting HCC initiation and progression.	[89]
miR-182	It activates the BCL2 gene to inhibit tumor cell apoptosis and also activates the Wnt/β-catenin pathway, promoting initiation, progression, invasion and cell metastasis.	[90,91]

### 3.3. Role of Long Non-Coding RNAs

During HCV infection, both viral replication and activation of interferon (IFN) signaling pathways promote the transcriptional induction of several cellular lncRNAs. Viral proteins and viral replication can activate transcription factors such as MYC, SP1, NRF2, and HIF1, which in turn modulate the expression of additional lncRNAs. Dysregulation of these transcripts in HCV-infected cells may exert proviral or antiviral effects by functioning as positive or negative regulators of the IFN response. As a result, lncRNAs may contribute to the development of liver fibrosis, cirrhosis, and HCV-induced hepatocellular carcinoma, or they may protect infected cells from these outcomes [92]. Table 2 summarizes selected lncRNAs that are dysregulated in HCV-infected cells and their respective functions.

In an experimental model, normal and malignant HCV-infected liver cells treated with IFN alpha exhibited increased expression of the lncRNA IFI6, which activated the expression of the interferon-stimulated gene IFI6 and inhibited viral replication. This antiviral activity was independent of the Janus kinase signal transducer and the JAK STAT transcriptional activation pathway. Overexpression of a truncated spatial domain or mutant form of lncRNA IFI6 suppressed IFI6 expression and increased HCV replication. These findings indicate that lncRNA IFI6 enhances antiviral innate immunity by activating the promoter and modifying histones of the IFI16 gene through its spatial domain [93].

LINC01189 is downregulated in chronically HCV-infected cells and in hepatocellular carcinoma. Forced expression of LINC01189 reduces tumor cell proliferation and chemoresistance to 5-fluorouracil. Hsa-miR-155-5p was identified as a downstream target of LINC01189 in HCC, and forced expression of hsa-miR-155-5p abolished the inhibitory effects of LINC01189 on cancer cell growth. These findings indicate that LINC01189 suppresses HCV-induced hepatocellular carcinoma by acting as a sponge for hsa-miR-155-5p, thereby reducing tumorigenicity [94]. By neutralizing the activity of hsa-miR-155-5p, LINC01189 counters the activation of oncogenic signaling pathways, including MAPK-associated proliferative signaling, and restores regulatory mechanisms that limit tumor cell proliferation, differentiation, and survival. Mechanistically, LINC01189 functions as a tumor suppressor by sequestering hsa-miR-155-5p and preventing it from inhibiting anti-proliferative gene expression.

Overexpression of NEAT1 and AKT2 promotes proliferation and invasion of hepatocellular carcinoma cells, inhibits apoptosis, and increases the invasive capacity of malignant cells. Opposite effects occur in NEAT1 knockdown models and following downregulation of AKT2, both of which reduce proliferation and invasion and increase apoptosis. Overexpression of AKT2 abolishes the inhibitory effects observed in the absence of NEAT1. Forced expression of miRNA 22 3p inhibits NEAT1 function, whereas inhibition of miRNA 22 3p restores NEAT1 activity. Both NEAT1 and AKT2 were identified as direct targets of miRNA 22 3p. These findings demonstrate that NEAT1 promotes hepatocellular carcinoma, both in vitro and in vivo, by downregulating miRNA 22 3p and preventing it from suppressing AKT2 expression [95,96].

The lncRNA UCA1 is upregulated in HCV-infected cells in a time- and dose-dependent manner and increases the expression of interferon-stimulated genes that reduce viral replication. UCA1 functions as a sponge for miR 145 5p, which normally inhibits expression of the cytokine gene SOCS7, a negative regulator of interferon signaling. By suppressing the activity of miR 145 5p, UCA1 increases SOCS7 expression, and the resulting protein product inhibits interferon responses and thereby facilitates viral. These findings indicate that under normal physiological conditions, miR-145-5p inhibits HCV replication by preventing SOCS7-mediated suppression of interferon production.

Another study demonstrated that NEAT1 also targets miR-5p, whose function is to downregulate the oncogene BGH3. miR 155-5p expression decreases following HCV infection, whereas BGH3 expression increases. High BGH3 expression is also observed in HCV-induced hepatocellular carcinoma and correlates with elevated NEAT1 levels. Suppression of NEAT1 increases miR-25-5p expression and reduces BGH3 expression, confirming their inverse regulatory relationship [83]. These findings indicate that NEAT1 acts as a sponge for miR-21-5p, preventing it from exerting its tumor suppressor function by repressing BGH3 oncogene expression.

A study investigated the relationship between the lncRNAs HOTTIP, H19, and HOTAIR and miRNA 152, as well as the role of these interactions in the progression of HCV-induced liver lesions to hepatocellular carcinoma. Sixty-five patients with chronic liver disease without HCC, thirty-eight patients with chronic liver disease with HCC, and thirty healthy volunteers were analyzed. The findings showed a consistent inverse correlation between the two lncRNAs and miRNA 152 as the disease progressed to malignant stages in patients chronically infected with HCV genotype 4 who subsequently developed cirrhosis and hepatocellular carcinoma [88]. These observations suggest that these transcripts act as sponges for miRNA 152 and prevent its inhibitory action on the Wnt1 signaling pathway, thereby promoting tumor initiation and progression.

Another study demonstrated that miRNA 152 has a crucial protective role in regulating cell proliferation by inhibiting the Wnt1 signaling pathway, which is implicated in the initiation of hepatocellular carcinoma. Neutralization of this protective function of miRNA 152 is mediated by the HCV capsid protein, which negatively regulates miRNA 152 expression and activates the Wnt1 pathway, thereby inducing tumor cell proliferation [97].

The relationship between the lncRNA HULC, which is highly expressed in HCV-induced hepatocellular carcinoma, and miR 372 was examined in forty patients with HCV-positive HCC, forty patients infected only with HCV, and twenty healthy controls. HULC expression levels were significantly elevated in patients with HCC compared with controls, whereas miR 372 expression was reduced and its target, PRKACB, was markedly upregulated [98]. These findings suggest that HULC acts as a sponge for miR-372 and prevents it from suppressing PRKACB, which promotes cell proliferation and supports the initiation and progression of hepatocellular carcinoma.

The lncRNA LINC00152 is upregulated in hepatocellular carcinoma, and serum levels of this transcript are higher in patients with HCC than in individuals with normal liver tissue. Elevated circulating levels of LINC00152 are an independent predictor of poor prognosis in HCC [99]. A higher LINC00152 to GAS5 ratio correlates significantly with increased mortality risk in patients with HCV-associated HCC. GAS5 inhibits expression of the viral gene encoding NS3, thereby reducing viral replication [100]. These findings suggest that LINC00152 promotes HCC progression, whereas GAS5 functions as a tumor suppressor.

Another study showed that LINC00152 promotes HCV-induced carcinogenesis by activating genes involved in cell cycle regulation, proliferation, epithelial–mesenchymal transition, and tumor invasion through modulation of several signaling pathways, including mTOR and EMT. It also decreases expression of essential epithelial markers such as EpCAM and E-cadherin [101]. Conversely, GAS5 suppresses HCV replication by interacting with the NS3 protein and inhibiting its functional activity, thereby reducing viral load and inflammation [102].

HCV infection reduces Linc Pint expression in hepatocytes through interaction with the C/EBP beta binding protein. Forced expression of Linc Pint increases IFN alpha and IFN beta expression in HCV-infected hepatocytes by activating the IFN alpha 14 promoter. Proteomic analysis demonstrated that Linc Pint binds to DDX24, facilitating the interaction of RIP1 with IRF7 in the interferon signaling pathway. These findings indicate that Linc Pint functions as a positive regulator of innate immune responses, particularly interferon signaling [103]. Thus, HCV-mediated suppression of Linc Pint appears to constitute an immune evasion strategy used by the virus to reduce host antiviral defenses.

DLEU2 and SNHG16 expression is upregulated in hepatocellular carcinoma compared with nontumor cirrhotic tissue. LINC00662 expression is also significantly elevated in HCC relative to cirrhotic tissue, although it shows no significant association with patient survival [104]. These findings suggest that LINC00662 may increase the risk of progression from HCV-associated liver injury to hepatocellular carcinoma, but it does not appear to influence overall disease-related mortality.

**Table 2:** List of selected lncRNAs cited in this review, categorized according to the functions they perform in HCV-infected liver cells and in hepatocellular carcinoma.

**LncRNA**	**Protective Function Against the Development of HCC**	**References**
lncRNA-IFI6	Increase the expression of the interferon-stimulating gene IF16 inhibiting HCV replication.	[93]
LINC01189	It works by sponging hsa-miR-155-5p preventing it from activating the Wnt/β-catenin pathway that leads to tumorigenicity in HCV-infected cells.	[94]
GAS5	Inhibits the expression of the viral gene of the NS3 protein, reducing HCV replication.	[100,102]
Linc-Pint	It activates the IFN-α14 promoter and increases the expression of IFN-α and IFN-β, but this action is inhibited in HCV-infected hepatocytes.	[103]
**LncRNA**	**Inducing function of HCC initiation and progression**	**References**
NEAT1	Downregulates miR-22-3p preventing suppression of Map3k12 and Sox9, allowing activation of NF-κB and AKT2 pathways and increasing inflammation and cell survival.	[95]
NEAT1	Acts as a miR-9-5 p sponge, preventing it from exerting its tumor suppressor action by repressing the BGH3 oncogene.	[83]
HOTTIP, H19 and HOTAIR	They act as a miR-152 sponge, preventing it from exerting its inhibitory action on the Wnt1 pathway involved in the initiation of HCC.	[88]
LINC00152	Activates signaling pathways, including mTOR and EMT, reducing the expression of key proteins such as EpCAM and E-cadherin, activating proliferation and mobility of tumor cells	[99,101]

### 3.4. Role of Circular Non-Coding RNAs

Circular RNAs (circRNAs) constitute a class of noncoding RNAs ubiquitously expressed in eukaryotic cells during post-transcriptional processing. They possess covalently closed 5 prime and 3 prime termini and display cell-specific and tissue-specific expression patterns that vary according to developmental stage. CircRNAs act as sponges for miRNAs and proteins, thereby regulating gene expression and modulating alternative pre-mRNA splicing, and some circRNAs can encode proteins. Dysregulated circRNA expression is associated with pathological states and various human diseases, although circRNAs may also induce antiviral immune responses. In vitro generated circRNAs activate RIG-I-mediated innate immune pathways, providing protection against viral infections [105].

CircRNAs derived from nuclear retrosplicing of pre-mRNAs have been implicated in altering the functions of virus-infected cells [106]. They may be encoded by both host cells and viruses, are highly stable, and can sequester proteins and miRNAs to modulate intracellular pathways [107]. RNA viruses such as HCV can generate self-derived viral circRNAs (vcircRNAs). Bioinformatic analysis of HCV genomic RNA indicates that it can fragment into hundreds of vcircRNAs, with more than a dozen amplified through a rolling circle mechanism. Those containing internal ribosome entry sites may be translated into proteins with proviral activity. At least two abundant untranslated vcircRNAs increase viral RNA copy number [106,107].

Hepatocytes contain numerous circRNA species, some upregulated and others downregulated during infection, exhibiting either proviral or antiviral activities. CircPSD3 is markedly upregulated and significantly increases viral RNA abundance in HCV-infected cells. It binds to the eIF4A3 factor, which modulates the nonsense-mediated decay pathway. Unexpectedly, circPSD3 enhances viral RNA amplification at a post-translational level, whereas eIF4A3 exerts antiviral NMD activity [108]. Certain HCV-encoded vcircRNAs contain ribosome entry sites and are translated into proteins with proviral effects. They may also bind miR 122, potentiating its essential role in positively regulating the HCV life cycle [106,108]. These observations indicate that viral circRNAs may be translated into proteins and can synergize with untranslated vcircRNAs to enhance viral replication.

Circ ITCH expression is elevated in patients with hepatocellular carcinoma compared with healthy controls and correlates with increased AST and ALT levels [109]. This suggests that liver-derived circITCH contributes to HCV-associated pathogenesis by promoting liver injury and progression to HCC. CircITCH may facilitate HCC progression by acting as a sponge for miR-224-5p, thereby preventing its inhibitory effect on MafF, a transcription factor involved in oncogene activation and tumor growth [110].

Conversely, a circITCH variant (hsa_circ_0001141) exhibits tumor-suppressive activity. Its overexpression inhibits proliferation and invasion and promotes apoptosis in HCV-induced HCC cells in vitro and in vivo, whereas its suppression has the opposite effect [111]. This circITCH isoform functions as a sponge for miR 184, preventing its carcinogenic effects in chronically HCV-infected cells. Forced expression of miR-184 induces cell cycle dysregulation by reducing expression of the tumor suppressor SOX7, which normally downregulates the oncogene c-MYC. Loss of SOX7 activity permits c-MYC overexpression and drives the initiation and progression of HCC [89].

Circ SERPINA3 expression is elevated in hepatocellular carcinoma and suppresses the antitumor activity of miR 944, reducing patient survival by approximately one year. MiR-944 negatively regulates MDM2, and its downregulation results in increased oxidative stress and enhanced metastatic potential in HCC. Reduced miR-944 expression promotes HCV-induced carcinogenesis by increasing MDM2 abundance and decreasing E-cadherin levels. These findings indicate that circSERPINA3 functions as a sponge for miR-944, inhibiting its tumor-suppressive role and increasing MDM2 expression, which promotes degradation of the tumor suppressor p53 [107].

CircRNA 0000502 binds miR 124, and circ 0001955 binds miR 145 5p; these interactions represent regulatory mechanisms observed in HCC that influence disease progression and distinguish tumor from non-tumor hepatic tissue [112]. In HCV-induced hepatocellular carcinoma, circRNA 0000502 sequesters miR 124, neutralizing its tumor-suppressive function and promoting proliferation, migration, and metastasis [113]. HCV suppresses miR 124 expression, whereas forced expression reduces AQP3 levels and inhibits tumor progression. CircHIPK3 is upregulated in HCC and increases AQP3 expression, thereby enhancing proliferation and migration. Upregulated circRNA 0000502 silences miR 124, preventing suppression of AQP3 and promoting tumorigenesis [84].

Circ 0001955, TRAF6, and MAPK11 are upregulated in hepatocellular carcinoma, whereas miR 145 5p and miR 516a 5p are downregulated. Suppression of circ0001955 reduces tumor growth in vitro and in vivo, whereas its overexpression has protumorigenic effects. Circ0001955 functions as a sponge for miR-145-5p, which normally inhibits proliferation, migration, and invasion and promotes apoptosis by targeting ARF6 and SPATS2. MiR 516a 5p targets TRAF6 and MAPK11 [85,86]. Binding of circ0001955 to miR-145-5p abolishes its tumor suppressor activity, thereby derepressing ARF6 and SPATS2. Binding to miR-516a-5p prevents downregulation of TRAF6 and MAPK11, which in turn activates NF-kappa B and MAPK pathways [85]. Collectively, these mechanisms allow circ0001955 to restore the carcinogenic activity of ARF6, SPATS2, TRAF6, and MAPK11 in chronically HCV-infected hepatocytes, promoting the initiation and progression of hepatocellular carcinoma.

SPATS2 participates in cell cycle progression and immune regulation and is a key driver of HCC progression. TRAF6 recruits and activates TAK1 and IKK to stimulate NF-kappa B and MAPK signaling pathways, enhancing inflammation and tumor cell survival. MAPK11 plays an essential role in MAPK signaling by promoting cellular proliferation, differentiation, and resistance to apoptosis [114].

MiR 145 5p also inhibits SPATS2 expression, whose protein product promotes proliferation, metastasis, and invasion in multiple cancers, including hepatocellular carcinoma. Under physiological conditions, miR 145 5p upregulates p21 and p27, which are negative regulators of the cell cycle that induce cell cycle arrest, promote DNA repair, reduce mutation rates, and inhibit proliferation, migration, and invasion [87].

CCK-8, BrdU, and Transwell assays demonstrated that the HCV NS3 protein activates genes associated with tumor cell migration. NS3 positively regulates circ 0001175, which functions as a sponge for miR 130a 5p, eliminating its protective regulation of p53 through the MDM4 pathway. MDM4 induces p53 degradation, thereby promoting HCV-induced carcinogenesis [115]. These findings suggest that NS3 increases circ0001175 expression, which silences miR-130a-5p and activates MDM4-mediated p53 degradation.

Low expression of hsa circ 0005986 and circ 102166 correlates with advanced hepatocellular carcinoma and poor patient survival [115]. These circRNAs likely act as tumor suppressors by sequestering miR 182, preventing activation of C1DN1, which favors HCV replication, and inhibiting oncogenic pathways that promote cell proliferation, resistance to apoptosis, and tumor cell motility [90]. MiR 182 also promotes tumorigenesis by activating BCL2, an antiapoptotic protein essential for HCC progression, and by stimulating Wnt beta catenin signaling [91].

**Figure 2:**
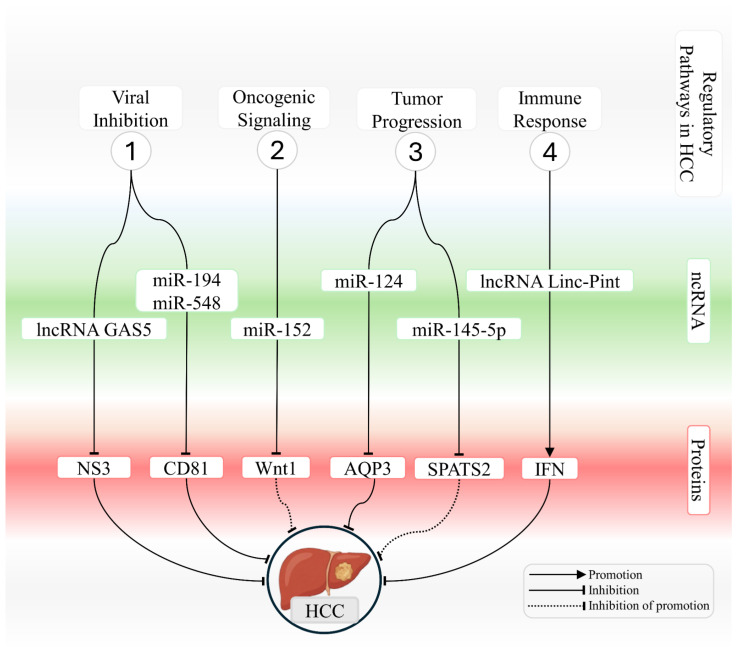
Integrated network of ncRNAs that modulate viral, oncogenic, and immune-related processes in HCV-associated hepatocellular carcinoma. The schematic illustrates key mechanisms regulated by microRNAs and long noncoding RNAs during hepatocarcinogenesis associated with HCV infection. The figure summarizes four functional modules. (1) Modulation of viral infection, in which lncRNA GAS5 and miR 194 and miR 548 repress essential targets required for viral entry and replication, including NS3 and CD81, thereby reducing cellular permissiveness to HCV. (2) Regulation of oncogenic signaling, where miR 152 suppresses the Wnt and Wnt1 axis and limits transcriptional activation that promotes proliferation. (3) Control of tumor progression, in which miR-124 and miR-145-5p regulate AQP3 and SPATS2, respectively, modulating proliferation, cell motility, and survival pathways. (4) Modulation of immune responses, in which lncRNA Linc Pint enhances interferon induction and strengthens antiviral and antitumor activity. Interactions are represented as promotion, inhibition, or inhibition of promotion, as indicated in the figure, outlining the regulatory architecture that links ncRNAs to protein targets and signaling pathways that either restrain or facilitate HCV-driven hepatocarcinogenesis.

Table 3 summarizes circRNAs that are dysregulated in HCV-infected liver cells and describes their oncogenic or protective functions in hepatocellular carcinoma. Figure 2 provides an integrated schematic representation of ncRNA-mediated interactions in chronically HCV-infected hepatocytes, highlighting pathways that either counteract or promote the development of hepatocellular carcinoma.

## 4. Conclusions

Despite substantial reductions in prevalence and incidence over recent decades, driven by major therapeutic advances, HCV infection remains a significant global public health challenge. Chronic hepatitis C is a leading cause of liver cirrhosis and hepatocellular carcinoma and continues to be a major contributor to liver disease-related mortality. Complete eradication of the virus remains an unmet objective. The marked genetic variability of HCV, coupled with its highly efficient immune evasion strategies, has hindered the development of a safe and effective vaccine.

The World Health Organization has set a target for 2030 to eliminate hepatitis C as a global public health threat by reducing the incidence of new infections by 90 percent and mortality by 65 percent. Advancing our understanding of the molecular mechanisms underlying gene expression dysregulation in chronically HCV-infected cells, together with the development of increasingly precise antiviral therapies, represents a promising route toward achieving this goal. Furthermore, deeper insights into the molecular basis of HCV-associated diseases may enable precision medicine approaches that facilitate personalized therapeutic strategies in the future.

Within this context, elucidating the mechanisms of action of ncRNAs involved in the pathogenesis of HCV-associated liver disease is essential for overcoming the numerous critical barriers that currently impede clinical translation. A major challenge lies in achieving precise modulation of these transcripts in vivo, given the complexity of their interactions with DNA, mRNA, proteins, and intracellular signaling pathways. Additional limitations include dosage considerations and the potential for unpredictable off-target effects resulting from the extensive range of genetic targets influenced by these molecules.

Another major challenge involves ensuring the stability of these molecules in the bloodstream and achieving their selective delivery to cancer cells while avoiding unintended effects on healthy tissues. In this context, ncRNAs, including microRNAs, long noncoding RNAs, and circular RNAs, show strong potential as noninvasive biomarkers not only for diagnosis and prognosis but also for the early detection of HCV-associated hepatocellular carcinoma. Their tissue specificity, dynamic expression patterns during disease progression, and measurable presence in liquid biopsies further strengthen their biomarker value. Nevertheless, rigorous validation of individual candidates and combined panels remains essential, since their involvement in multiple regulatory pathways may affect diagnostic specificity. Looking ahead, the incorporation of multiomics approaches such as transcriptomics, epigenomics, proteomics, and metabolomics will be crucial for fully elucidating ncRNA-mediated networks and for advancing the development of reliable early detection biomarkers and therapeutic targets.

## Figures and Tables

**Table 3:** List of circRNA types categorized according to the functions they perform in liver cells infected with HCV.

**CircRNAs**	**Protective Function Against the Development of HCC**	**References**
CircITCH (has-circ-0001141)	Acts as a sponge for miR-184, preventing it from performing its function in the progression of HCC, which is to inhibit the tumor suppressor SOX7.	[89,111]
Hsa_circ_0005986 e circ-102,166	They act as a sponge for miR-182, preventing it from exercising its function of activating oncogenic pathways that lead to proliferation, apoptosis and cell motility of HCC cells.	[90,91]
**CircRNAs**	**Inducing function of HCC initiation and progression**	**References**
CircSERPINA3	It acts as a miR-944 sponge, abolishing its tumor suppressor function with an increase in MDM2 and a reduction in the activity of p53 and E-cadherin.	[107]
Circ-ITCH	It acts by activating miR-224-5p, which activates MafF, a transcription factor that plays a role in activating the expression of oncogenes.	[110]
CircRNA_0000502 e circHIPK3	Both act as a miR-124 sponge, neutralizing its tumor suppressor action in HCC by increasing AQP3 expression.	[84]
Circ_0001955	It acts as a sponge for miR-145-5p and prevents its tumor suppressor action.	[86]
Circ_0001175	Viral NS3 upregulates circ_0001175 which acts as a miR-130a-5p sponge, abolishing its p53 activating function and modulating the MDM4 pathway	[115]

## Data Availability

No new data were created or analyzed in this study. Data sharing is not applicable to this article.
